# Preferences for peer-reviewed versus other publication sources: a survey of general dentists in the National Dental PBRN

**DOI:** 10.1186/s13012-019-0854-x

**Published:** 2019-03-01

**Authors:** Julia Melkers, Diana Hicks, Kimberley R. Isett, Dorota T. Kopycka-Kedzierawski, Gregg H. Gilbert, Simone Rosenblum, Vanessa Burton, Rahma Mungia, Michael J. Melkers, George Ford, the National Dental PBRN Collaborative Group

**Affiliations:** 1 School of Public Policy, Georgia Institute of Technology, Atlanta, GA USA; 2 University of Rochester Medical Center, Rochester, NY USA; 3 School of Dentistry, University of Alabama at Birmingham, Birmingham, AL USA; 4 HealthPartners Dental Group, Minneapolis, MN USA; 5 University of Texas Health Science Center at San Antonio, School of Dentistry, San Antonio, TX USA; 6 Hanover, USA; 7 Lawrenceville, USA

**Keywords:** Information preferences, General dentist, Peer-reviewed, Journal articles, Survey, Advanced training, FAGD

## Abstract

**Background:**

Medical professionals have access to a broad range of resources to address clinical information needs. While much attention is given to new sources of data such as those available on the internet, it is less clear how clinicians choose between peer-reviewed research literature and other publication-based sources. This analysis distinguishes between possible drivers of publication type preference (namely, practice setting, advanced training, professional development experiences). Dentists enrolled in the National Dental Practice-Based Research Network (PBRN) are the population for this study. Theories of human and intellectual capital and institutional logics theory are used to understand how advanced training and other clinical experiences may explain the choices that dentists make when faced with clinical questions.

**Methods:**

An online questionnaire was implemented with general dentists in the US National Dental PBRN. A series of logistic and Ordinary Least Squares (OLS) regression models were used to explain the use of peer-reviewed and other publications. Measures of knowledge-based human capital distinctions (advanced clinical training and research engagement, advanced professional status, personal motivation for professional advancement) were used to explain preferences for research literature as a clinical resource.

**Results:**

General dentists with advanced training, as well as those with a skill advancement motivation, show a preference for peer-reviewed materials. General dentists who have been practicing longer tend to favor other dental publications, preferring those sources as a resource when faced with clinical challenges. Human capital and professional motivation distinguish the information preferences among general dentists. Further, these factors explain more variance in use of peer-reviewed materials than practice setting does. Few differences by demographic groups were evident.

**Conclusions:**

Results point to a distinct variation in the general dentistry professional community. Advanced training among general dentists, as well as the types of procedures typically conducted in their practice, distinguishes their information preferences from other general dentists, including those with more years of clinical experience.

## Background

By improving knowledge on how information is accessed by clinical professionals, decisions about implementing information dissemination strategies can be made to more effectively get information into the hands of clinicians. Studies of information-seeking behavior in clinical settings have increasingly focused on the modality of knowledge access (print, internet, and interpersonal sources) [1–11], where peer-reviewed journals that provide access to clinical trial research findings, case reports, and systematic reviews, remain relevant to the delivery of new clinical knowledge in the practice setting. The focus on how clinicians access these information sources, however, does not adequately address differences in the content nor selectivity of the information sources, leaving a gap in our understanding of what explains preferences for high-quality peer-reviewed materials over other published materials. A natural next step is to establish if preferences for different types of publication resources vary and to look for factors that explain this variation. Our interest is not whether dentists access information online or not, nor how they seek information from colleagues. Instead, we ask, what determines these preferences?

In a profession where advanced credentials do not necessarily mean a change in position (general dentists typically remain general dentists), how are preferences for information sources affected by the pathways dentists take for ongoing knowledge development relevant to their practice? We focus on general dentists because they comprise the largest group of dental clinicians, work in a range of different practice settings, and provide an array of dental services [12, 13], suggesting different information needs within this population. With 83% of youth and 64% of adults having a dental examination in the past year [14], understanding the information-seeking behavior of the general dentist may inform ways to increase the uptake of evidence-based research for clinical decision-making in an understudied clinical service delivery area, and could provide insights for other classes of clinical professionals.

### Human capital pathways and information preferences in dentistry

While dentists’ information-seeking behavior is presumably driven by a complex set of factors, including problem complexity, information type, and convenience [15], we argue that personal factors also shape that behavior. Human capital [16–19] and institutional logics [20] theories are social-economic and organizational theories that help to frame the variation in information source preferences. “Institutional logic” refers to the “individual norms, assumptions, beliefs, and preferences” that form as a result of the norms and contexts of the setting in which an individual works or operates. In dentistry [21], studies have addressed the relationship between logics of medical professionalism that value clinical care with business-oriented commercial logics that focus on efficiency and profit in practice management. It is the differences in these “logics” (medical and business) that we argue are embodied in individual clinician choices about building their human capital/knowledge that may shape information preferences [22].

Human capital refers to the acquired knowledge, skills, judgment, and other traits of individuals, obtained through formal and informal education and experiences [23, 24]. Health professionals are trained to rely to a large extent on their own professional expertise and tacit knowledge in treating patient needs [25] based on the foundation of professional training and related credentials. Clinicians are also expected (and often required) to formally remain “current” on materials, procedures, disease, and other factors relevant for effective clinical practice [26], typically through continuing education (CE) [27]. Considerable variability in the rigor, intensity, and reputation of CE sources, coupled with clinician choice of what to attend [28, 29], means that how clinicians obtain CE is highly variable. In addition to clinical values and business sensibilities, we expect that dentists who choose a rigorous and formal pathway to knowledge development via rigorous CE and/or further credentialing will also demonstrate an “academic logic” [30, 31]. It is the differences in these “logics” (medical and business), that we argue are embodied in individual clinician choices about building their human capital/knowledge, that may shape their information preferences. *We hypothesize that clinicians who are personally motivated for demanding professional advancement will prefer peer-reviewed resources more than will those clinicians without that experience.*

Human capital is also acquired through experience. Clinicians who have accumulated knowledge [16] through their own career may have less interest or need to navigate complex and time-consuming written materials [2, 4, 7, 32, 33], which may enable them to more easily discern the utility and relevance from other curated sources. *We therefore hypothesize that clinicians with knowledge based on accumulated experience will align more with a “medical professionalism” logic and show a lower preference for more difficult to access (peer-reviewed) materials and a higher preference for curated publications (magazines and other publications)*. Based on the above, we expect that knowledge and the motivation to develop that human capital both matter for information preferences among general dentists.

## Methods

Our analysis is based on an online questionnaire of dentists and hygienists in the National Dental PBRN, a consortium of dental practitioners and organizations focused on improving the scientific basis for clinical decision-making [13, 34, 35]. The questionnaire was designed by a team of social scientists and dental professionals to gather data about where and how dental practitioners access clinically-relevant evidence. The protocol was approved by the central and regional IRB offices of the National Dental PBRN and was administered online from August to December 2016. PBRN members were alerted to the survey by the PBRN regional coordinators in each US region and were then invited to complete the survey by the study PI via US postal service letter followed by a personal e-mail. Reminders were sent monthly. Consistent with survey design principles [36], details of the survey purpose were kept to a general level in order to reduce possible bias or priming of the respondents. The survey was pretested [36, 37] using cognitive pretesting with clinicians to address understandability, coverage, and readability of the survey, and a field pretest addressed response patterns. Minor adjustments to the survey content and order were made as a result of these pretests.

The sampling frame of 3106 practicing dentists and hygienists included all network members who were practicing dentists or hygienists but whose primary affiliation was not an academic institution. The sample was stratified by National Dental PBRN geographic regions and oversampled for groups under-represented in the dental profession (female dentists, male hygienists, racial/ethnic minorities). A final adjusted response rate of 58% (*N* = 1722) was achieved after data cleaning (removing partial responses or individuals not in clinical practice). Given our interests, we limited our focus here to the 1191 general dentists in our sample. General practitioners deliver the majority of dental care, work in a range of practice settings, and provide a range of services [12, 38]. The representativeness of our respondent group was compared to the National Dental PBRN and to the population of dentists and hygienists in the USA (Table 1). On average, our sample is representative of the National Dental PBRN but does differ from the national population of dentists and hygienists in a few demographic aspects. Our deliberate oversampling of female dentists and racial/ethnic minorities for analytical reasons reflects a purposive over-representation of individuals in these groups. The PBRN is least representative of very young dentists (under the age of 35). The questionnaire data were supplemented with data from the PBRN enrollment questionnaire [39], providing additional demographic and practice information used in this analysis.

**Table 1:** National Dental PBRN study representativeness (PBRN and National) dentists and practice characteristics (data limited to dentists only, omitting dental specialists and hygienists)

	Study sample	PBRN^i^	BLS^ii^	ADA^iii^
All practitioners (dentists, specialists, hygienists)	*n* = 1722	*N* = 5000	*N* = 373,000	NA
Dentists only	*n* = 1360	*N* = 3553	*N* = 196,000	*N* = 195,722
Variables:	%	%	%	%
Female	35.1	28.3	25.9	29.8
Black	5.5	4.2	NA	NA
Asian	11.5	9.5	16.8	NA
Hispanic	6.7	6.1	8.6	NA
Age under 35 ^a, b, c^	8.0	6.6	NA	15.91
35 to 44 ^a, b^	25.9	22.2	NA	22.79
45 to 54	21.2	21.2	NA	21.52
55 to 64 ^a, b^	30.4	31.7	NA	24.82
65 and older ^b, c^	13.5	17.0	NA	14.96
General practice ^a, b, c^	87.6	74.9	NA	79.1
Oral and maxillofacial surgery ^b, c^	1.5	1.7	NA	3.9
Endodontics ^a, b^	4.1	3.0	NA	2.8
Periodontics ^b, c^	4.3	4.0	NA	2.8
Prosthodontics	1.5	1.8	NA	1.8
Public health dentistry* ^a, b, c^	6.0	3.7	NA	0.4
Practice characteristics				
Percent of patients on private insurance	60.1	59.6	NA	64.7
Percent of patients on public insurance	17.08	16.31	NA	8.7
Percent of patients without coverage	23.37	24.5	NA	26.6
	Mean	Mean	Mean	Mean
Patient wait time for treatment appointment (days)	7.48	7.57	NA	5.2
Patient wait time in waiting room (minutes)	8.53	8.49	NA	7.4
Patients per week**(number)	42.14	52.8	NA	76.5

^i^Data from PBRN enrollment questionnaire sent in April 2016

^ii^Using 2015 data from Bureau of Labor Statistics (https://www.bls.gov/cps/aa2015/cpsaat11.htm)

^iii^Using 2015 data from American Dental Association (https://www.ada.org/en/science-research/health-policyinstitute/data-center/dental-practice)

* Sample is statistically significantly different from BLS population (*P* < .05)

**PBRN is statistically significantly different from BLS population (*P* < .05)

^a^Sample is statistically significantly different from network (*P* < .05)

^b^Sample is statistically significantly different from ADA population (*P* < .05)

^c^PBRN is statistically significantly different from ADA population (*P* < .05)

The specific variables included in our analysis are discussed below with operationalization details listed in Table 2 and descriptive statistics in Table 3.

- *Information source use and preferences.* Survey respondents were asked “Which of the following journals or publications do you typically consult in instances when you do not have sufficient information to treat or advise a patient? (These may be print or online version.)” Respondents were offered a list of 11 items that included peer-reviewed journals and other dental and medical publications, as well as an “other” option. Journals were selected based on readership patterns identified in prior studies [10, 40] (reflecting those most used) as well as consultation with the clinicians on our study team. First, we created a binary variable for whether respondents indicated that they consulted at least one peer-reviewed dental publication. We then created three variables based on a sum of sources to measure the range of sources: peer-reviewed journals, other publications, and overall (the sum of the previous two categories). We conceptualized this as a range of core sources relevant to general dentistry, rather than a count because we did not offer a comprehensive list to respondents. Finally, we created a proportional measure to examine the mix of publications consulted (E-I Index) [41]. This measure, which ranges from − 1 to + 1, indicates whether some dentists lean more toward peer-reviewed journals (− 1) or other publications (+ 1). Zero indicates an equal distribution between the two types of publications. Missing values (130) for this variable exist due to (a) the 104 general dentists who indicated in an earlier question that they do not turn to dental journals at all when faced with a clinical scenario that they do not know how to treat and (b) 26 respondents who did not indicate a specific journal in a follow-on question, either in the close-ended option or in the fill-in-the-blank “other.”
- *Clinical complexity and practice setting.* Because information needs may vary with practice setting, we included data that differentiated practice contexts. Respondents were asked about the frequency (never, rarely, and routinely) with which they conducted a range of dental procedures. Based on review and input from the dental clinicians on our study team, we categorized these as either “standard” (common procedures in general dentistry) or “complex” (procedures typically performed by dental specialists but may also performed by general dentists) procedures (detailed procedures are listed in in Table 1). While clinicians may perform these complex procedures from time to time, we were most interested in whether a dentist performed them on a regular basis, which would then distinguish these practices from those that are limited to more standard procedures. We then created two count variables, the first reflecting a count of the number of standard procedures performed on a routine basis and the second reflecting the number of complex procedures performed on a routine basis. These two count variables therefore capture the range of procedures performed in a clinician’s office. Regarding other practice context, we also included data on overall patient load, whether the dentist is a solo practitioner, whether they practice full- or part-time, and their rural/urban location (based on county). This allows us to account for accessible interpersonal resources available to our set of general dentists [16, 42].
- *Knowledge development*. We focused on two types of knowledge in our analysis: formal and informal/experiential, where formal knowledge development reflects educational degrees and other credentials and informal knowledge is based on experience. For general dentists, 1- or 2-year residency programs, referred to either as a General Practice Residency (GPR, usually hospital-based) or as an Advanced Education in General Dentistry (AEGD; usually based at an academic institution) program, are advanced options, both involving a rigorous number of hours, as is the AGD master program, which requires 1100 h of continuing education credit in a wide range of general dentistry topics. Few dentists (30% of respondents) met the criteria for advanced training in general dentistry (i.e., did a GPR or an AEGD program), and these respondents were coded as “1.” The two experiential development variables included whether the respondent (a) ever led a clinical study, worked on a clinical study team, or personally applied for research funding (based on a survey question and coded as “1” if they had done any of these) [25] and (b) has tacit knowledge accumulated through years in practice (based on time since dental school graduation), recognized as a legitimate learning source in clinical and other professions [11, 25, 26, 28] and coded as a continuous variable.
- *PBRN skill motivation*. Individuals differ in motivation to affiliate with a community of practice or research network [43, 44]. Given our interest in understanding differences in information-seeking behavior, we wanted to isolate whether individuals were more motivated by knowledge and skill acquisition, as compared to relational/networking reasons, in their decision to affiliate with the National Dental PBRN. In response to the question “How important were the following reasons in your decision to enroll in the PBRN?” **r**espondents were asked to select from a range of seven individual items. These included networking with current or new colleagues, CE credits, taking part in clinical studies, and the two items used for this measure (skill development and knowledge development) to indicate its importance (not at all important, somewhat important, important, very important). PBRN skill motivation was coded as “1” if respondents indicated PBRN engagement for the purposes of knowledge and skill development rather than networking (indicating another mechanism for dentists to expand their knowledge foundation relevant to clinical dentistry) was “very important.”
- *Demographics.* Under-represented populations in many professions have different experiences from the dominant social group (in this case, white males). We included measures from the National Dental PBRN enrollment questionnaire for female dentists and for under-represented minority dentists (Hispanic, African American, or Native American) as standard control measures to identify any differences by demographic groups.

**Table 2:** Variable description

Variable	Survey question
Dependent Variables	
Consults dental peer-reviewed journals	Consults at least one of the following peer-reviewed dental journals: Journal of the American Dental Association, General Dentistry, Compendium of Continuing Education in Dentistry, other dental peer-reviewed (coded from open-ended responses).
Range of peer-reviewed journals consulted	Which of the following journals or publications do you typically consult in instances when you do not have sufficient information to treat or advise patients? Sum of items: Journal of the American Dental Association, General Dentistry, Compendium of Continuing Education in Dentistry, other dental peer-reviewed (coded from open-ended responses).
Range of other published sources consulted	Which of the following journals or publications do you typically consult in instances when you do not have sufficient information to treat or advise patients? Sum of items: American Dental Association News, Dentistry Today, Inside Dentistry, other non-peer-reviewed (coded from open-ended responses)
Proportional mix of peer-reviewed journals and other published sources	E-I Index formula: (range of other published sources consulted − range of peer-reviewed journals consulted)/(range of other published sources consulted + range of peer-reviewed journals consulted)
Independent variables	
Clinical setting	
Solo practitioner	How many of the following, including yourself, work in your practice? (response = 1) (Dentist may work in private or other practice setting)
Dentist practices full-time	Do you work: full-time (1); part-time (0)
Dentist practices in one office	Do you work in: one office (1); multiple offices (0)
Number of complex procedures routinely conducted	Please indicate the frequency with which you personally perform the following procedures in a typical month (not at all, occasionally, routinely). Sum of respondent responses indicating that the following procedures were conducted routinely: (implants (prosthetic and surgical procedures for implants), periodontal therapy (surgical), endodontic therapy (surgical), endodontic therapy (molars), orthodontic treatment)
Number of standard procedures routinely conducted	Please indicate the frequency with which you personally perform the following procedures in a typical month (not at all, occasionally, routinely). Sum of respondent responses indicating that the following procedures were conducted routinely: (non-implant restorative (amalgams, composites, crowns, veneers, bridges, posts, foundations, etc), removable prosthetics (full and partial dentures), extractions (surgical and non-surgical), periodontal therapy (non-surgical, includes scaling/root planning), procedures for esthetic reasons only (composites, crowns, veneers, etc.), endodontic therapy (anteriors/pre-molars)).
Number of patients per week	On average, how many patients (both operative and hygiene combined) are seen overall in your practice per week? (1 = less than 21 patients, 2 = 21–30 patients, 3 = 31–40 patients, 4 = 41–50 patients, 5 = 51–60 patients, 6 = 61 patients or more)
Practice in rural area	Office located in county not adjacent to county with major metropolitan center
Human capital/PBRN skills motivation	
Advanced training	Completed either: FAGD, MAGD, GPR, AEGD, other advanced training (open-ended)
Research experience	Since you graduated from dental school, have you: (responded to at least one) (led a clinical study, worked on a clinical study team, personally applied for research funding)
PBRN skill motive	How important were the following reasons in your decision to enroll in the National Dental PBRN? (Learn about new techniques or activities relevant to: (answered “very important” to one of the following: learn about new techniques or activities relevant to my clinical practice, learn about new techniques or activities relevant to my practice management))
Professional age	Years since dental school graduation
Demographics	
Female dentist	Are you: (male, female)
Under-represented minority dentist	Are you: (binary variable for affirmative responses for: Hispanic, African American, or Native American)

**Table 3:** Descriptive statistics

	Percentage or mean	SD	Min	Max	*N*
Dependent variables					
	%				
Consults peer-reviewed dental journals (Y/N)	87	0.33	0	1	1191
Consults other published dental sources (Y/N)	58	0.49	0	1	1191
	Mean				
Range of all sources consulted	2.78	1.71	0	7	1191
Range of peer-reviewed journal sources consulted	1.74	1.00	0	4	1191
Range of other published sources consulted	1.05	1.09	0	4	1191
	E-I Index				
Proportional mix of peer-reviewed Journals and other published sources	−0.37	0.53	−1	1	1061
Independent Variables					
Clinical Setting					
	Mean				
Range of complex procedures routinely conducted	0.66	0.85	0	4	1191
Range of standard procedures routinely conducted	3.49	1.49	0	6	1191
Range of patients per week (category)	5.07	1.41	1	6	1189
	%				
Solo practitioner	47	0.50	0	1	1191
Dentist practices full-time	88	0.32	0	1	1191
Dentist practices in one office only	81	0.38	0	1	1191
Human/intellectual capital motivation					
	%				
Advanced training	31	0.46	0	1	1191
Research experience	40	0.49	0	1	1191
PBRN skill motive	65	0.48	0	1	1191
	Mean				
Professional age (years since dental school)	24.2	12.32	1	66	1155
Demographics					
	%				
Female dentist	37	0.48	0	1	1191
Under-represented minority dentist	13	0.34	0	1	1191

Analysis involved both descriptive and regression analysis using Stata statistical software (version 14.2). For the descriptive analysis, we examined means and standard deviations, as well as correlation matrices (Table 4). We used logistic regression models to explain the likelihood that a general dentist would consult a peer-reviewed journal when faced with a clinical challenge, given that the dependent variable is binary. For the remaining models, we ran a series of Ordinary Least Squares (OLS) regression models, appropriate for continuous dependent variables, to test the knowledge source effects on the range of peer-reviewed journals, other publications, and overall publication sources consulted (variables are summarized in Table 1). For all models, we present the results in stepwise order, where the practice and knowledge variables are introduced sequentially to better isolate and understand the effects of each of these variables on information preferences. For example, we ran models with only practice characteristics and human capital variables separately and then together. Sampling weights were used due to the stratified sampling procedures used.

**Table 4:** Pairwise Pearson correlations: independent variables in regression models

		1	2	3	4	5	6	7	8	9	10	11	12	13	14	15	16	17	18
Consults Peer-Reviewed Dental Journals	1	1.000																	
Consults Other Published Dental Sources	2	0.333*	1.000																
Range of All Sources Consulted	3	0.565*	0.698*	1.000															
Range of Peer-Reviewed Journal Sources Consulted	4	0.664*	0.298*	0.800*	1.000														
Range of Other Published Sources Consulted	5	0.278*	0.822*	0.835*	0.338*	1.000													
Proportional Mix of Peer-Reviewed/Other Sources	6	−0.379	0.877*	0.499*	−0.201	0.839*	1.000												
Professional Age	7	0.029	0.114*	0.139*	0.093*	0.132*	0.091*	1.000											
Advanced Training	8	−0.049	0.028	0.104*	0.183*	−0.005	−0.089	0.299*	1.000										
Research Experience	9	−0.028	0.018	0.056	0.0664*	0.027	−0.036	0.177*	0.196*	1.000									
PBRN Skill Motive	10	0.071*	0.113*	0.123*	0.088*	0.112*	0.076*	−0.077	0.014	−0.066	1.000								
Female	11	−0.027	−0.042	− 0.075	−0.077	− 0.048	− 0.014	− 0.296	−0.114*	− 0.086	0.033	1.000							
Underrepresented Minority	12	−0.039	0.025	−0.005	− 0.041	0.030	0.050	−0.061	0.011	0.007	0.096*	0.088*	1.000						
Solo Practitioner	13	−0.027	0.093*	0.089*	0.047	0.096*	0.108*	0.179*	0.095*	−0.014	0.006	−0.100	− 0.039	1.000					
Full Time Dentist	14	0.008	0.011	0.061*	0.0654*	0.037	0.009	− 0.133	0.009	−0.016	0.078*	−0.140	0.041	0.019	1.000				
Rural Practice	15	0.019	0.062*	0.054	0.013	0.072*	0.061*	−0.032	0.013	0.028	0.028	−0.012	−0.035	− 0.009	−0.033	1.000			
Number of Complex Procedures Routinely Conducted	16	−0.006	0.044	0.078*	0.0782*	0.051	0.032	0.1745*	0.231*	0.113*	0.020	−0.212	− 0.026	0.243*	0.057*	−0.010	1.000		
Number of Standard Procedures Routinely Conducted	17	0.079*	0.097*	0.132*	0.1105*	0.1059*	0.059	0.0605*	0.101*	0.056	0.1201*	−0.092	0.0712*	0.161*	0.066*	0.038	0.453*	1.000	
Number of patients per week	18	−0.036	0.028	0.000	0.025	−0.022	−0.045	− 0.108	−0.062*	− 0.017	0.004	− 0.075	− 0.050	− 0.099	0.139*	− 0.026	0.009	0.007	1.000

## Results

### Descriptive analysis

The descriptive statistics of the survey results are presented in Table 2. Of the 1191 general dentists who replied to the survey, most were male (63%), and white (68%), reflecting the characteristics of the dentist population. The general dentist respondents varied in their formal and experiential access to human capital as measured in this study. First, 31% of the respondents reported having formal advanced training beyond that required for CE. In terms of experiential intellectual capital development, almost half (40%) reported having had personal experience in clinical research, including having led a clinical study, worked on a clinical study team, or personally applied for research funding. In terms of professional age, respondents graduated from dental school an average of 24 years ago, but the sample also included very new and quite seasoned/near retirement dentists. Note that one criterion was that all survey respondents are still practicing clinical dentistry.

Roughly half of the general dentists were solo practitioners (47%), and on average, reported seeing over 60 patients per week. Respondents also varied in the types of clinical procedures routinely conducted in their practices. Not surprisingly, standard procedures were more common than complex. Respondents on average routinely conducted three of the five standard procedures included in the enrollment questionnaire and about one complex procedure on a routine basis.

**Fig. 1:**
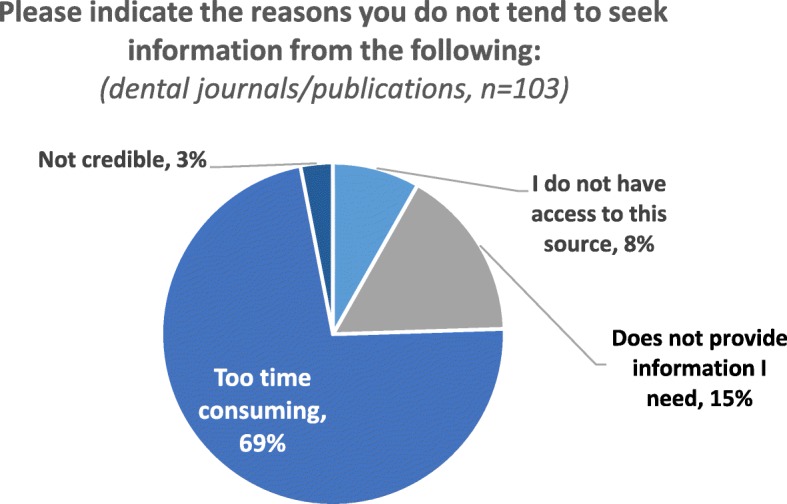
Rationale for not using journal publications

Further, we found some variation among the general dentists for our dependent variables of interest—the preference for different publication sources. As expected, the vast majority (87%) of respondents reported that they would typically consult a peer-reviewed dental journal when they needed information to advise or treat a patient and more than half (58%) reported that they would typically consult another type of publication in this case (respondents could check all that apply). On average, general dentists reported consulting three out of six specifically named peer-reviewed journals and a mixed set of other sources, with peer-reviewed sources being more prevalent than other published sources. For the small set of general dentists who did not report turning to peer-reviewed publications, access was not a barrier for most. Time and relevance instead were offered as reasons for not turning to peer-reviewed materials in clinical uncertainty (Fig. 1).

### Regression analysis: preference for peer-reviewed sources

Our first question asks how practice setting and the human/intellectual capital of general dentists explain their preference for consulting a peer-reviewed journal when they are faced with a clinical scenario where they lack sufficient information to treat or advise a patient. To address this question, we constructed a stepwise logistic regression model (Table 5). Odds ratios are provided to identify the odds of whether a general dentist with any of the characteristics captured by our independent variables is more or less likely to consult one or more of the peer-reviewed dental journals common to general dental practice. Variable inflation factor (VIF) statistics for each of the models were also run and showed no multicollinearity effects in any of the models.

**Table 5:** Stepwise logistic regressions. General dentist consults reer-reviewed journals in dentistry. “Which of the following journals or publications do you typically consult in instances when you do not have sufficient information to treat or advise patients?” (consults at least one)

	Odds ratios			
	Basic model	Practice context model	Human capital model	Full model
Demographics				
Female dentist	0.860	0.894	0.903	0.940
Under-represented minority dentist	0.725	0.699	0.736	0.706
Clinical setting				
Solo practitioner	0.828	0.818	0.830	0.863
Dentist practices full-time	1.138	1.027	1.149	1.066
Dentist practices in one office only	0.955	0.978	0.945	0.955
Rural practice		1.325		1.220
Number of patients per week		1.070		1.081
Number of complex procedures routinely conducted		0.850		0.796
Number of standard procedures routinely conducted		1.237**		1.22**
Human/intellectual capital motivation				
Advanced training			1.338	1.410
Research experience			1.150	1.158
PBRN skill motive			1.707**	1.58*
Professional age (years since dental school)			1.004	1.005
Constant	8.211***	3.445*	4.587**	1.929
Observations	1191	1189	1155	1153

***p < 0.001, **p < 0.01, *p < 0.05

Prob > *χ*^2^ = 0.0074

Log pseudo likelihood = − 499.09086, pseudo *R*^2^ = 0.0315

In the limited logistic regression model, results showed no significant relationship of various demographic or practice characteristics and the likelihood of a general dentist to consult peer-reviewed journals when faced with a clinical question. When aspects of their clinical practice are added to the model, the results showed that an increase in the number of routine procedures regularly conducted resulted in a 20% higher likelihood that a dentist will consult a peer-reviewed dental journal when faced wtih a clinical uncertainty. Notably conducting complex procedures on a routine basis had no significant effect. Regarding human/intellectual capital factors, the results of the full model showed that dentists with advanced training or personal experience with research are no more likely to consult a peer-reviewed source than their colleagues without these experiences. However, those dentists who are seeking skill development through the National Dental PBRN are 70% more likely to consult peer-reviewed journals than their colleagues who joined the PBRN for other reasons. These odds diminished slightly when combined in the full model (Odds ratio = 1.58 for those with PBRN skill motivation, or 58% more likely) but generally held.

### Regression analysis: range of peer-reviewed and other sources consulted

While there was little variation in whether dentists consult peer-reviewed journals under conditions of uncertainty as shown above, there was more variation in the breadth and mix of the resources they consult. Tables 5 and 6 show the results of the weighted regression models that examined the extent to which the same set of independent variables explains variation in the number and the mix of peer-reviewed journals and other published sources. Models were stepped to examine human capital and practice effects in more limited models and then to observe the results when these variables were combined. First, to examine the factors that explain the breadth of overall as well as peer-reviewed journals and other resources consulted in cases of clinical uncertainty, Table 6 shows few significant relationships between practice settings and information preference. General dentists working in a practice that conducts a broader range of standard clinical procedures on a routine basis read a wider range of sources overall as well as both peer-reviewed journals and other sources, holding in all models (coefficient ranging from 0.06 to 0.17 depending on the model). Other aspects of practice setting, such as regular complex procedures or overall patient load, did not statistically correspond to preferences for peer-reviewed sources.

**Table 6:** Weighted regression results: practice setting and human and intellectual capital in explaining publication preferences (Ordinary Least Squares)

	All sources				Peer-reviewed sources				Other dental sources			
	Basic model	Practice context model	Human capital model	Full model	Basic model	Practice context model	Human capital model	Full model	Basic model	Practice context model	Human capital model	Full model
Female dentist	− 0.244*	− 0.215*	− 0.102	− 0.089	− 0.141*	− 0.110	− 0.095	− 0.079	− 0.102	− 0.105	− 0.007	− 0.010
	(0.105)	(0.107)	(0.110)	(0.111)	(0.062)	(0.063)	(0.066)	(0.066)	(0.067)	(0.068)	(0.069)	(0.069)
Under-represented minority dentist	− 0.008	− 0.038	− 0.085	− 0.104	− 0.114	− 0.125	− 0.166	−0.169	0.106	0.086	0.081	0.065
	(0.154)	(0.155)	(0.152)	(0.153)	(0.088)	(0.088)	(0.086)	(0.086)	(0.097)	(0.099)	(0.100)	(0.101)
Solo practitioner	0.391**	0.296*	0.272*	0.228	0.109	0.051	0.053	0.032	0.282***	0.244**	0.219**	0.196*
	(0.124)	(0.131)	(0.127)	(0.132)	(0.074)	(0.078)	(0.076)	(0.079)	(0.077)	(0.081)	(0.079)	(0.083)
Dentist practices full-time	0.253	0.222	0.327	0.294	0.180	0.152	0.175	0.149	0.073	0.070	0.152	0.145
	(0.172)	(0.178)	(0.167)	(0.172)	(0.097)	(0.100)	(0.098)	(0.100)	(0.101)	(0.104)	(0.099)	(0.101)
Dentist practices in one office only	0.035	0.082	0.045	0.074	0.046	0.061	0.045	0.050	− 0.011	0.021	− 0.001	0.024
	(0.138)	(0.136)	(0.135)	(0.134)	(0.083)	(0.083)	(0.080)	(0.081)	(0.085)	(0.083)	(0.084)	(0.083)
Rural practice		0.578		0.488		0.106		0.071		0.473*		0.416*
		(0.342)		(0.360)		(0.179)		(0.191)		(0.196)		(0.198)
Number of complex procedures routinely conducted		0.013		− 0.036		0.050		0.004		− 0.038		− 0.040
		(0.070)		(0.072)		(0.042)		(0.044)		(0.044)		(0.045)
Number of standard procedures routinely conducted		0.176***		0.146**		0.077**		0.060*		0.099***		0.087**
		(0.047)		(0.046)		(0.027)		(0.027)		(0.029)		(0.029)
Number of patients per week		0.002		0.026		0.014		0.026		− 0.013		0.000
		(0.041)		(0.041)		(0.023)		(0.023)		(0.025)		(0.025)
Professional age (years since dental school)			0.016***	0.016***			0.002	0.002			0.014***	0.014***
			(0.005)	(0.005)			(0.003)	(0.003)			(0.003)	(0.003)
Advanced training			0.208	0.195			0.368***	0.356***			− 0.160*	− 0.161*
			(0.124)	(0.126)			(0.072)	(0.074)			(0.080)	(0.081)
Research experience			0.128	0.114			0.085	0.078			0.043	0.036
			(0.113)	(0.113)			(0.065)	(0.065)			(0.071)	(0.071)
PBRN skill motive			0.563***	0.505***			0.257***	0.232***			0.306***	0.273***
			(0.112)	(0.114)			(0.066)	(0.067)			(0.070)	(0.070)
Constant	2.328***	1.841***	1.435***	0.976**	1.531***	1.249***	1.214***	0.956***	0.797***	0.593**	0.221	0.019
	(0.222)	(0.321)	(0.258)	(0.361)	(0.128)	(0.182)	(0.158)	(0.212)	(0.135)	(0.193)	(0.156)	(0.217)
Observations	1191	1189	1155	1153	1191	1189	1155	1153	1191	1189	1155	1153
R-squared	0.016	0.034	0.059	0.070	0.013	0.026	0.062	0.068	0.013	0.029	0.051	0.062

Robust standard errors in parentheses

***p < 0.001, **p < 0.01, *p < 0.05

When the human and intellectual capital variables were introduced, we observed important effects. General dentists with advanced training (AGD Fellow and other) were more likely to broadly consult the peer-reviewed literature (coefficient 0.368, *p* < 0.001), but this training was significantly and negatively associated with the breadth of other non-peer-reviewed publications sources consulted overall (− 0.161, *p* < 0.05). Experiential intellectual capital in terms of years of experience was significant (*p* < 0.001) (although with small coefficients) in explaining the overall breadth of resources consulted, as well as the other published sources, but was not significantly related to the likelihood of consulting peer-reviewed journals. Clinician interest in increasing their knowledge and skill capital was also important. Motivation for skill development in the form of a rationale for joining the PBRN was positively and significantly associated (*p* < 0.001) with the breadth of sources consulted overall (coefficient 0.505) in both the peer-reviewed journal (coefficient 0.232) and other publication contexts (coefficient 0.273). Having participated or having led clinical research has no significant effect on source preferences.

Factors reflecting possible isolation were significantly associated with consulting other published knowledge sources (Table 6). General dentists practicing in a rural setting and solo practitioners were significantly more likely to consult a broader range of other sources (coefficient 0.416 and 0.196, *p* < 0.05 respectively in the full model when we control for all variables), while this was not the case for peer-reviewed journals. We also found that female dentists consulted a narrower range of published sources overall, but this effect disappeared when other factors were introduced to the model, showing that gender factors were minimally related to source preferences.

### Regression analysis: mix of peer-reviewed and other sources consulted

Finally, we addressed the proportional mix of sources consulted in order to identify whether general dentists tended to prefer more peer-reviewed or non-peer reviewed material across all of the publication sources they tended to consult. Which factors explained the mix of preferred sources? The final models addressed the balance of publication sources consulted using the E-I index [45]. Table 7 presents the results of the weighted regression model taking into account clinical setting and human and intellectual capital in explaining the proportion of publication preferences consulted by general dentists, i.e., taking into account reliance on peer-reviewed journals versus other sources consulted. Again, an E-I index closer to − 1 means that respondents proportionally consulted more peer-reviewed sources, while closer to + 1 means that they consulted more of the other publication sources.

**Table 7:** Weighted regression results: practice setting and human and intellectual capital in explaining proportion of publication preferences (E-I Index)

	Proportion of peer-reviewed journals and other published sources consulted							
	Basic model		Practice context model		Human capital model		Full model	
	Coeff	SE	Coeff	SE	Coeff	SE	Coeff	SE
Demographics								
Female dentist	− 0.016	(0.035)	− 0.020	(0.035)	0.01	(0.036)	0.012	(0.037)
Under-represented minority dentist	0.086	(0.050)	0.083	(0.050)	0.084	(0.052)	0.082	(0.052)
Practice setting								
Solo practitioner	0.158***	(0.040)	0.155***	(0.042)	0.140***	(0.042)	0.133**	(0.043)
Dentist practices full-time	− 0.007	(0.049)	0.001	(0.049)	0.035	(0.050)	0.039	(0.051)
Dentist practices in one office only	− 0.035	(0.042)	− 0.025	(0.043)	− 0.027	(0.042)	− 0.017	(0.043)
Rural practice			0.177*	(0.075)			0.158*	(0.074)
Number of complex procedures routinely conducted			− 0.018	(0.022)			− 0.007	(0.022)
Number of standard procedures routinely conducted			0.011	(0.012)			0.011	(0.012)
Number of patients per week			− 0.010	(0.012)			− 0.007	(0.012)
Human/intellectual capital motivation								
Professional age (years since dental school)					0.006***	(0.001)	0.006***	(0.001)
Advanced training					− 0.156***	(0.037)	− 0.154***	(0.037)
Research experience					− 0.036	(0.034)	− 0.039	(0.034)
PBRN skill motive					0.087*	(0.035)	0.081*	(0.035)
Constant	−0.468***	(0.064)	−0.456***	(0.095)	− 0.655***	(0.082)	− 0.65***	(0.112)
Observations	1061		1061		1030		1030	
R-squared	0.015		0.020		0.050		0.054	

Robust standard errors in parentheses

***p < 0.001, **p < 0.01, *p < 0.05

Looking first at the practice setting model, solo practitioners and general dentists in rural practices tended to consult more sources other than peer-reviewed journals, with results holding when the remaining variables were introduced (coefficients 0.177 and 0.158, *p* < 0.05 respectively). Finally, for our central interest in the sources of human/intellectual capital, results were as expected. Having advanced training showed a statistically significant preference for a somewhat higher proportion of peer-reviewed journals (with the largest coefficient of − 0.154, *p* < 0.001), while increased professional age as well as PBRN skills motivation showed a statistically significant preference for a balanced proportion of publication sources with coefficients close to zero (0.006, *p* < 0.001 and 0.081, *p* < 0.05). No significant results were found based on other practice characteristics or demographics.

## Discussion

Our purpose was to address the extent to which general dentists favor peer-reviewed sources over other publications when they are faced with a clinical question that requires a search for information beyond their own knowledge. While other research has focused heavily on the modality of information [26, 46–48], our attention to the distinction between peer-reviewed and other sources highlights the value placed on the evidence-based pyramid and peer-reviewed publications in the dental profession, regardless of whether they are accessed online or in print form. Not surprisingly, the vast majority (87%) of general dentists in our study acknowledged consulting peer-reviewed journals for clinical needs, leaving a small subset of dentists who do not. However, for the majority who do consult peer-reviewed sources, our results show that beyond this general measure there is considerable variation in the breadth and mix of publications that general dentists consult. As a caveat, these results do not raise a value judgment, but instead, shed light on source preferences and patterns among general dentists.

Perhaps, the most important finding of our work was in the ability to distinguish subgroups within the profession of general dentists, particularly related to advanced knowledge acquisition and knowledge-seeking motivation. Our primary interest was in how clinical knowledge developed professionally or through formal continuing education resources shaped information preferences. Like other knowledge-based professions, dentists initially acquire the foundation for this knowledge through professional training (dental school) and then build on it throughout their careers via both formal investments in their human capital (continuing education or formal residency programs) and informally by continually adapting their knowledge through experiential learning processes of daily practice [28, 29, 49, 50] gaining perspective and experience on successful and failed solutions to clinical challenges [50], as well as information sources relevant to clinical practice. We expected variation in these knowledge foundations to shape information preferences, at least in part.

Our findings show that, indeed, general dentists are anything but general and differ considerably in their preference for peer-reviewed publications. While the number of general dentists who do not consult peer-reviewed journal sources to address clinical questions is small, we expected that dentists with more intensive postgraduate training would favor these sources. We find that this does not explain whether a general dentist consults peer-reviewed materials at all, but we do find that this is the case when it comes to breadth of peer-reviewed and proportion of sources that they consult. Most importantly, the ways in which dentists develop their professional knowledge matter. Dentists who opt to pursue formal advanced training as a general dentist (in other words, not advanced training to become a specialist) prefer peer-reviewed journals as a resource, while those who have gained their knowledge through years of cumulative practice instead favor publications other than peer-reviewed journals. The latter is also aligned with prior work, raising concerns about the knowledge behavior and acquisition of older dentists [51] and suggestions that experiential clinical practice may cause clinicians to adapt knowledge but not necessarily add new research/scientific knowledge [50]. Interestingly, getting involved in clinical research studies had no effect on preferences for peer-reviewed or other publications in our sample, suggesting that limited research experiences may not shape these preferences.

Through our analysis, it seems that we may have identified a distinction between the academically inclined and the practitioner general dentist. As dentists professionally mature through years of practice, we find a preference for curated and typically more-condensed materials. Dentists who choose a formal pathway to advanced clinical knowledge development complement their clinical professionalism with an “academic logic” [30, 31], develop comfort and norms for certain materials, and through this intensive process, enhance their absorptive capacity or the ability to identify and apply the academic literature to their own needs [49]. This is important because as Cohen and Leventhal noted in their seminal piece, intensive exposure to diverse knowledge not only enhances the ability to assimilate new knowledge, but also has the added value of enabling a person to connect knowledge to different applications or problems [49]. Studies have shown that while logics may be conflicting, they may also interact [21]. It is the combination of these “logics” that shapes the preferences, norms, and decisions of these general dentists throughout their professional career and that we observe in our analysis [22].

Similarly, the knowledge- and skills-based motivation to affiliate with the PBRN also seems to identify the general dentist who is eager for more resources. Underscoring the personal nature of information priorities and interests, results show that these dentists who deliberately seek skills and knowledge through the PBRN consulted more of both the peer-reviewed journals as well as other publications (although with preference for the latter). This is consistent with information-seeking models of physicians in PBRN [34, 52, 53] and suggests a potentially different type of dentist than those who have affiliated with the PBRN for other reasons (such as those interested in networking and meeting colleagues).

Acknowledging the variation in the practice settings of general dentists, we also accounted for whether different aspects of the dentists’ work environment played a role in source preferences. Here, we found that different characteristics matter, some in surprising ways. Regarding the nature of their practice work, our expectation was that regularly conducting complex procedures may require greater upkeep of research-based clinical knowledge (and therefore favor peer-reviewed publications). However, the results did not support this expectation; the number of routine complex clinical procedures was not significantly associated with the number of sources, peer-reviewed or otherwise. However, working in an office that had a higher number of standard procedures was significantly associated with preference for a broader set of overall sources, peer-reviewed and other publications. This suggests that it is not the complexity of procedures that causes general dentists to consult materials, but rather the breadth of routinized procedures [54]. Given that many general dentists perform a broad set of procedures, this circumstance may motivate general dentists to keep current on a diverse set of clinical services that they provide.

Our results show that whether dentists practice full- or part-time, or in multiple offices, has no bearing on their preference for peer-reviewed journals or other sources. Based on social capital models of information acquisition [16, 42], this is not necessarily surprising. Since prior studies that have shown preference for interpersonal sources over others, having daily access to other dental colleagues may mean that seeking print resources (peer-reviewed or not) is not a priority, nor the most efficient or effective way to locate information. It is interesting, however, that solo practitioners and dentists in rural practices (who may be more isolated from colleagues outside of their office) consult a broader range of other published sources and also show a preference for proportionally more of these sources over peer-reviewed publications. This is consistent with the notion that journals and other print materials are not a substitute for having a colleague in the practice to readily consult. For example, a recent study of Danish primary care physicians observed that solo physicians are less likely to use colleagues as an information source, while also manifesting no differences in use of other sources [55]. Finally, we found almost no significant results across traditionally under-represented demographic groups (women and under-represented minorities).

### Strengths, limitations, and implications

Our findings are important for implementation research and practice because they highlight the information preferences used by one class of medical professionals, dentists, demonstrating that different types of dissemination pathways are reaching distinctly different sets of clinicians. A strength of our work is that the data include details on specific sources beyond the simple binary measure of whether a clinician ever consults the peer-reviewed literature. This binary operationalization is an overly simplistic view and is misleading in suggesting that all peer-reviewed journals are considered accessible and timely sources of information for clinicians. Our results are able to disentangle this to some degree. We are also able to examine not only the use, but also the proportional mix of those sources. Like any information source, it is not a zero-sum game and this approach allowed us to identify preferences in a more nuanced way.

There are also some important limitations to our work. We focused on the journals most read in the dental community, and a larger study of the corpus of dental publications could reveal other source preferences than we are able to reach with our current data. We also do not know the specific types of clinical issues that trigger the need to go beyond one’s expertise to seek out information in published materials. While we excluded dentists who are full-time faculty, we were not able to identify part-time or informal ties with academic institutions that might influence source preferences. Social desirability bias may exist, where dentists with more advanced training may feel compelled to indicate that they consult the peer-reviewed literature. We also do not know whether or how the nature of the information in those sources promoted uptake and implementation in the clinical setting for a particular type of issue, nor do we know how or why advanced training inspires or socializes dentists to give more weight to the peer-reviewed materials. Finally, we do not know enough about the skill versus credential or other motivations of general dentists to pursue advanced continuing education in general dentistry.

Our research has implications for future study, as well as for clinical practice. Future research may address the ways that social capital and access to colleagues shape reliance on or preferences for different types of publications including for rural dentists who are more geographically isolated. Also worth exploring is how residency training programs and advanced continuing education programs shape a general dentist’s professionalism and intellectual capital and why we observe the preferences for peer reviewed materials. Most importantly, it would be informative to know how and under what clinical circumstances general dentists raise questions and how those questions are addressed [52, 56], building on work in medicine [52, 57–59].

## Conclusion

General dentists vary in their preference for peer-reviewed literature when addressing clinical challenges. Dentists who are motivated to gain additional skills, and those who have achieved high-level postgraduate certifications, rely on a broader set of information resources and show a distinct preference for peer-reviewed sources. Results suggest that absorptive capacity for rigorous peer-reviewed sources may be enhanced through intensive advanced training credentials.

## References

[1] Hughes B, Joshi I, Lemonde H, Wareham J (2009). Junior physician’s use of Web 2.0 for information seeking and medical education: a qualitative study. Int J Med Inform.

[2] Thompson C, McCaughan D, Cullum N. Research information in nurses’ clinical decision-making: what is useful? Adv Nurs. 2001; http://onlinelibrary.wiley.com/doi/10.1046/j.1365-2648.2001.01985.x/full. 10.1046/j.1365-2648.2001.01985.x 11686752

[3] Isham A, Bettiol S, Hoang H, Crocombe LA (2016). Systematic literature review of the information-seeking behavior of dentists in developed countries. J Dent Educ.

[4] McCaughan D, Thompson C, Cullum N, Sheldon T, Raynor P (2005). Nurse practitioner and practice nurses’ use of research information in clinical decision making: findings from an exploratory study. Fam Pract.

[5] Hicks D, Isett K, Melkers J (2017). Evolving dental media: implications for evidence-based dentistry. Int J Evidence-Based Pract Dent Hyg.

[6] Melkers J, Hicks D, Rosenblum S, Isett KR, Elliott J. Dental blogs, podcasts, and associated social media: descriptive mapping and analysis. J Med Internet Res. 2017;19. 10.2196/jmir.7868 PMC5553003 28747291

[7] Coumou HC, Meijman FJ (2006). How do primary care physicians seek answers to clinical questions? A literature review. J Med Libr Assoc.

[8] Bocchi J, Eastman JK, Swift CO (2004). Retaining the online learner: profile of students in an online MBA program and implications for teaching them. J Educ Bus.

[9] Strother EA, Lancaster DM, Gardiner J (1986). Information needs of practicing dentists. Bull Med Libr Assoc.

[10] Botello-Harbaum MT, Demko CA, Curro FA, Rindal DB, Collie D, Gilbert GH (2013). Information-seeking behaviors of dental practitioners in three practice-based research networks. J Dent Educ.

[11] Landry CF (2006). Work roles, tasks, and the information behavior of dentists. J Am Soc Inf Sci Technol.

[12] Gilbert GH, Gordan VV, Korelitz JJ, Fellows JL, Meyerowitz C, Oates TW (2015). Provision of specific dental procedures by general dentists in the National Dental Practice-Based Research Network: questionnaire findings. BMC Oral Health.

[13] Makhija SK, Gilbert GH, Rindal DB, Benjamin P, Richman JS, Pihlstrom DJ. Dentists in practice-based research networks have much in common with dentists at large: evidence from “the Dental PBRN”. Gen Dent. 2009;57:270-275. PMC2819020 19819818

[14] National Center for Health Statistics. Health, United States, 2015: With Special Feature on Racial and Ethnic Health Disparities. Heal United States, 2015 With Spec Featur Racial Ethn Health Disparities. 2016;:107. doi:76–641496. 27308685

[15] Case D, Given LM (2016). Looking for information: a survey of research on information seeking, needs, and behavior-Ch. 5. Looking for information: a survey of research on information seeking, needs, and behavior.

[16] Nahapiet J, Ghoshal S (1998). Social capital, intellectual capital, and the organizational advantage. Acad Manag Rev.

[17] Swart J (2006). Intellectual capital: disentangling an enigmatic concept. J Intellect Cap.

[18] Vale J, Branco MC, Ribeiro J (2016). Individual intellectual capital versus collective intellectual capital in a meta-organization. J Intellect Cap.

[19] Coleman JS. Social capital in the creation of human capital. Am J Sociol. 1988:95–120.

[20] Thornton PH, Ocasio W, Lounsbury M. The institutional logics perspective. In: Greenwood, R., Oliver, C., Lawrence, T. B., & Meyer RE, editor. The SAGE handbook of organizational institutionalism; SAGE publications Inc 2012. p. 99–128.

[21] Harris R, Holt R (2013). Interacting institutional logics in general dental practice. Soc Sci Med.

[22] Greenwood R, Díaz AM, Li SX, Lorente JC (2010). The multiplicity of institutional logics and the heterogeneity of organizational responses. Organ Sci.

[23] Sweetland SR (1996). Human capital theory: foundations of a field of inquiry. Rev Educ Res.

[24] Becker GS (1962). Investment in human capital: a theoretical analysis. J Polit Econ.

[25] Malterud K (2001). The art and science of clinical knowledge: evidence beyond measures and numbers. Lancet.

[26] Leckie GJ., Pettigrew KE., Sylvain C. Modeling the information seeking of professionals: a general model derived from research on engineers , health care professionals , and lawyers. Libr Q information, community, Policy 1996;66:161–193.

[27] Schleyer TKL, Dodell D (2005). Continuing dental education requirements for relicensure in the United States. J Am Dent Assoc.

[28] Eraut M (2000). Non-formal learning and tacit knowledge in professional work. Br J Educ Psychol.

[29] McGowan BS, Wasko M, Vartabedian BS, Miller RS, Freiherr DD, Abdolrasulnia M (2012). Understanding the factors that influence the adoption and meaningful use of social media by physicians to share medical information. J Med Internet Res.

[30] Fini R, Lacetera N. Different yokes for different folks: individual preferences, institutional logics, and the commercialization of academic research. In: Spanning the boundaries and disciplines: university technology commercialization in the idea age: Emerald Group Publishing; 2010. p. 1–25.

[31] Sauermann H, Stephan P (2013). Conflicting logics? A multidimensional view of industrial and academic science. Organ Sci.

[32] Spallek H, O’Donnell J, Clayton M, Anderson P, Krueger A (2010). Paradigm shift or annoying distraction: emerging implications of web 2.0 for clinical practice. Appl Clin Inform.

[33] Wahoush O, Banfield L (2014). Information literacy during entry to practice: information-seeking behaviors in student nurses and recent nurse graduates. Nurse Educ Today.

[34] Andrews JE, Pearce KA, Ireson C, Love MM (2005). Information-seeking behaviors of practitioners in a primary care practice-based research network (PBRN). J Med Libr Assoc..

[35] Gilbert GH, Williams OD, Korelitz JJ, Fellows JL, Gordan VV, Makhija SK (2013). Purpose, structure, and function of the United States National Dental Practice-Based Research Network. J Dent.

[36] Dillman DA, Smyth JD, Christian LM (2014). Internet, phone, mail, and mixed-mode survey: the tailored design method.

[37] Presser S, Couper MP, Lessler JT, Martin J, Rothgeb JM (2004). Methods for testing and evaluating survey questions. Public Opin Q.

[38] Brennan D, Spencer A (2005). The role of dentist, practice and patient factors in the provision of dental services. Community Dent Oral Epidemiol.

[39] The National Dental Practice-Based Research Network (2016). Rapid disruptions: understanding the dental information networks around alternative nicotine products and other clinical needs relevant to patient health and health.

[40] Funkhouser E, Agee BS, Gordan VV, Rindal DB, Fellows JL, Qvist V (2014). Use of online sources of information by dental practitioners: findings from the dental practice-based research network. J Public Health Dent.

[41] Borgatti SP, Everett MG (2000). Models of core/periphery structures. Soc Networks.

[42] Wasko MM, Faraj S (2005). Why should I share? Examining social capital and knowledge contribution in electronic networks of practice. MIS Q.

[43] Andrew N, Ferguson D, Wilkie G, Corcoran T, Simpson L (2009). Developing professional identity in nursing academics: the role of communities of practice. Nurse Educ Today.

[44] Fagnan LJ, Handley MA, Rollins N, Mold J (2010). Voices from left of the dial: reflections of practice-based researchers. J Am Board Fam Med.

[45] Krackhardt D, Stern RN (1988). Informal networks and organizational crises: an experimental simulation. Soc Psychol Q.

[46] Dawes M, Sampson U (2003). Knowledge management in clinical practice: a systematic review of information seeking behavior in physicians. Int J Med Inform.

[47] Straub-Morarend CL, Marshall TA, Holmes DC, Finkelstein MW (2011). Informational resources utilized in clinical decision making: common practices in dentistry. J Dent Educ.

[48] Iqbal A, Glenny A-M (2002). General dental practitioners’ knowledge of and attitudes towards the employment of dental therapists in general practice. Br Dent J.

[49] Cohen WM, Levinthal DA (1990). Absorptive capacity: a new perspective on learning and innovation. Adm Sci Q.

[50] Hurst D, Mickan S (2017). Describing knowledge encounters in healthcare: a mixed studies systematic review and development of a classification. Implement Sci.

[51] Sadowsky D, Kunzel C (1989). Professional life cycle changes and their effect on knowledge level of dental practitioners. Soc Sci Med.

[52] Del Fiol G, Workman TE, Gorman PN (2014). Clinical questions raised by clinicians at the point of care: a systematic review. JAMA Intern Med.

[53] Gorman PN, Yao P, Seshadri V (2004). Finding the answers in primary care: information seeking by rural and nonrural clinicians. Stud Health Technol Inform.

[54] Perrow C (1967). A framework for the comparative analysis of organizations. Am Sociol Rev.

[55] Le JV, Pedersen LB, Riisgaard H, Lykkegaard J, Nexøe J, Lemmergaard J (2016). Variation in general practitioners’ information-seeking behaviour–a cross-sectional study on the influence of gender, age and practice form. Scand J Prim Health Care.

[56] Brassil E, Gunn B, Shenoy AM, Blanchard R. Unanswered clinical questions: a survey of specialists and primary care providers. J Med Libr Assoc. 2017;105. 10.5195/JMLA.2017.101. 10.5195/jmla.2017.101 PMC5234458 28096740

[57] Alper BS, White DS, Ge B (2005). Physicians answer more clinical questions and change clinical decisions more often with synthesized evidence: a randomized trial in primary care. Ann Fam Med.

[58] Oscheroff J, Forsythe D, Buchanan B, Bankowitz R, Blumenfled B, Miller R (1991). Physician’s information needs: analysis of questions asked during clinical teaching. Ann Intern Med.

[59] Cook DA, Sorensen KJ, Wilkinson JM, Berger RA (2013). Barriers and decisions when answering clinical questions at the point of care. JAMA Intern Med.

